# Visual Experience Shapes Orthographic Representations in the Visual Word Form Area

**DOI:** 10.1177/0956797616657319

**Published:** 2016-07-20

**Authors:** Heinz Wimmer, Philipp Ludersdorfer, Fabio Richlan, Martin Kronbichler

**Affiliations:** 1 Centre for Cognitive Neuroscience, University of Salzburg; 2 Neuroscience Institute, Christian Doppler Clinic, Paracelsus Medical University Salzburg

**Keywords:** fMRI, neuroimaging, reading, visual word recognition, visual word form area, orthographic representations

## Abstract

Current neurocognitive research suggests that the efficiency of visual word recognition rests on abstract memory representations of written letters and words stored in the visual word form area (VWFA) in the left ventral occipitotemporal cortex. These representations are assumed to be invariant to visual characteristics such as font and case. In the present functional MRI study, we tested this assumption by presenting written words and varying the case format of the initial letter of German nouns (which are always capitalized) as well as German adjectives and adverbs (both usually in lowercase). As evident from a Word Type × Case Format interaction, activation in the VWFA was greater to words presented in unfamiliar case formats relative to familiar case formats. Our results suggest that neural representations of written words in the VWFA are not fully abstract and still contain information about the visual format in which words are most frequently perceived.

Competent readers are affected only to a minor extent by the specific appearance of written words (e.g., *brain* vs. *BRAIN*). The predominant view in the past decades of psychological research has been that the robustness of visual word recognition rests on abstract memory representations for letters and consequently also words (for a review, see Rayner, Pollatsek, Ashby, & Clifton, 2012). These representations are assumed to be insensitive to the specific visual attributes of a presented word stimulus, such as case, font, or retinal location.

The assumption that letters and words are represented abstractly has also been adopted by recent neuroscientific research. Electrophysiological studies, for example, have suggested the existence of an abstract letter-identification processing stage distinct from a preceding letter-form-identification stage (e.g., Carreiras, Perea, Gil-López, Mallouh, & Salillas, 2013). Neuroimaging studies suggest that visual word recognition relies on a hierarchy of increasingly larger and more abstract neural representations along the left ventral visual pathway (e.g., Dehaene, Cohen, Sigman, & Vinckier, 2005). Central to this account is the so-called *visual word form area* (VWFA; Cohen et al., 2000; Cohen et al., 2002) situated in the left ventral occipitotemporal cortex (vOT), which is assumed to host neural representations coding for abstract letters, letter sequences, and small words (Vinckier et al., 2007). Although the VWFA account is not uncontroversial (Price & Devlin, 2003, 2011), the general importance of this region for reading is evident from neuropsychological studies that have shown that damage to the left vOT causes a relatively isolated deficit in visual word recognition (e.g., Gaillard et al., 2006; Leff et al., 2001).

Evidence for the assumption that representations in the VWFA are abstract was initially provided by a study showing that activation in this region was invariant to the retinal location of presented words (Cohen et al., 2000). Of main importance, however, were functional MRI (fMRI) priming studies, which found repetition-suppression effects for words in the VWFA to be independent of case (Dehaene et al., 2004; Dehaene et al., 2001; see also Devlin, Jamison, Gonnerman, & Matthews, 2006). Subliminal primes presented in a case different from that of the target word (e.g., *car*-*CAR*) led to the same activation reduction in the VWFA, as did same-case primes (e.g., *CAR*-*CAR*) relative to different-word primes (e.g., *DOT*-*CAR*). These findings were taken to reflect that both types of primes preactivate abstract representations stored in the region (Dehaene et al., 2004).

However, several previous findings are difficult to reconcile with the assumption that letter and word representations in the VWFA are abstractions that do not include specific visual attributes. Burgund, Guo, and Aurbach (2009), for example, failed to find case-independent repetition suppression for letters in the VWFA (see also Gauthier et al., 2000, for similar findings using letters in different fonts). Doubts about abstract representations were also raised by studies that compared words presented in an unfamiliar mixed-case format (e.g., *mIxEd*) with words presented in a familiar format; these studies found increased VWFA activation in response to the unfamiliar format (Kronbichler et al., 2009; Xu et al., 2001). However, words presented in mixed case are also known to result in low-level visual-processing difficulties, such as lateral interference (e.g., misplaced uppercase letters interfering with neighboring lowercase letters; Mayall, Humphreys, & Olson, 1997). Consistent with such low-level difficulties, the findings of Xu et al. (2001) and Kronbichler et al. (2009) showed increased activation for mixed-case words not only in the VWFA but also in more posterior occipital regions. The increased VWFA response to mixed-case words therefore may have resulted from a downstream effect of the high activation in posterior regions.

The aim of the present fMRI study was to provide a more stringent test of whether representations in the VWFA are fully abstract or still contain information about the visual format in which words are most frequently perceived. To this end, we based our study on behavioral research that has shown that even minor deviations from the familiar visual format—such as presenting the initial letter of a word in an unfamiliar case—affect word-recognition speed (Jacobs, Nuerk, Graf, Braun, & Nazir, 2008; Peressotti, Cubelli, & Job, 2003). Following Jacobs et al. (2008), we presented German words with the initial letter in either uppercase or lowercase. The presented words were either nouns or nonnouns (i.e., adjectives and adverbs). Critically, while German nouns are always seen with initial capitalization (e.g., *Ball* [ball]), German adjectives and adverbs are most commonly seen in lowercase (e.g., *blau* [blue]). Adjectives and adverbs are capitalized only at the beginning of sentences and when used as nouns. By presenting both nouns and nonnouns, we were therefore able to manipulate case-format familiarity independently of physical case format (i.e., uppercase vs. lowercase). In addition, presenting the initial letters of nouns and nonnouns in an unfamiliar case does not pose unusual visual-processing demands, because—in contrast to the mixed-case formats used by previous studies—both formats are commonly used in German.

We expected that if representations in the VWFA are fully abstract (Dehaene et al., 2004; Dehaene et al., 2001), the present case-format manipulation should have no significant effect on activation in this region because recognition of both familiar and unfamiliar case formats should be supported by the same abstract representations. If, however, representations in the VWFA do contain information about the visual format in which words are most frequently perceived, the present case-deviant forms should violate these representations. This should result in an interactive effect of word type (nouns vs. nonnouns) and case format (uppercase vs. lowercase) on VWFA activation, with increased activation for words presented in unfamiliar case formats.

## Method

### Participants

Twenty-six German-speaking university students (13 female, 13 male) between the ages of 20 and 41 years (*M* = 29 years) participated in the study. All participants had normal or corrected-to-normal vision and reported no history of neurological or psychiatric disease or reading or spelling difficulties. All gave informed consent and were paid for participation. All methods conformed to the code of ethics of the World Medical Association (Declaration of Helsinki). According to the institutional guidelines of the University of Salzburg, there was no need for ethical approval because the present study was noninvasive and performed on healthy volunteers (https://online.uni-salzburg.at/plus_online/wbMitteilungsblaetter.display?pNr=98160). The sample size was determined on the basis of prior fMRI studies from our lab investigating similar effects in reading (Kronbichler et al., 2007; Kronbichler et al., 2009).

### Task and stimuli

For the in-scanner task, participants were instructed to indicate with a two-choice key press whether the presented stimulus was or was not an existing German word (i.e., a lexical decision task). All participants saw the same 384 items (half words, half pseudowords), but each participant saw the items in one of two pseudorandomized lists. Half of the word items were nouns, and the other half were adjectives and adverbs (i.e., nonnouns). Half of the items in each category (i.e., nouns, nonnouns, and pseudowords) were presented with the initial letter in uppercase, and the remainder were presented with the initial letter in lowercase. The case format of the first letter of each item varied between the two pseudorandomized lists. Counterbalancing the lists ensured that both forms were presented equally often. As shown in Table 1, pseudowords were roughly matched to words with respect to number of letters, bigram frequency (based on the CELEX database; Baayen, Piepenbrock, & van Rijn, 1993), and number of orthographic neighbors (i.e., Coltheart’s *N*) in order to prevent lexical decisions being made on the basis of any of those factors. The fact that nouns and nonnouns were not perfectly matched is of little relevance because we did not aim to compare word types. The critical manipulation concerned the case format of the initial letters, which for each item was changed from participant to participant. Hence, case format varied independently from item characteristics.

**Table 1.** Comparison of the Mean Characteristics of the Word and Pseudoword Items

	Words				Words vs. pseudowords	
Characteristic	Nouns	Nonnouns	All	Pseudowords	*t* (191)	*p*
Number of letters	4.4 (0.6)	4.5 (0.7)	4.5 (0.5)	4.5 (0.5)	< 1	> .250
Word frequency (per million)	165 (29)	263 (41)	214 (25)	—	—	—
Bigram frequency (per million)	11,625 (837)	13,530 (894)	12,578 (615)	11,001 (445)	1.24	.217
Number of orthographic neighbors	3.6 (0.3)	3.2 (0.3)	3.4 (0.2)	3.0 (0.2)	1.89	.058

Note: Standard deviations are given in parentheses.

The 384 items were presented in two experimental runs of 192 items each, with an equal number of words and pseudowords. Each item was displayed for 800 ms, with an interstimulus interval (ISI) of 2,100 ms, during which a fixation cross was shown. This stimulus onset asynchrony of 2,900 ms was not an integer of the repetition time of 2,000 ms (see fMRI Data Acquisition and Analysis), which enhanced the efficiency of the design by sampling the hemodynamic response at different time points. In addition to the items, 40 null events of 2,900-ms duration, during which only a fixation cross was presented, were included in each run. The null events were included to improve evaluation of stimulus-related activation relative to baseline.

Participants were familiarized with the lexical decision task outside the scanner. During scanning, visual stimuli were projected on a semitransparent screen by a video projector outside the scanner room. Participants used a magnetic-resonance-compatible response box, responding with the index finger (“yes”) and the middle finger (“no”) of their right hands. Stimulus delivery and response registration were controlled by Presentation software (Neurobehavioral Systems, Albany, CA).

### fMRI data acquisition and analysis

During each of the two functional-imaging runs, 340 images sensitive to blood-oxygen-level-dependent (BOLD) contrast were acquired with a T2*-weighted echo-planar imaging sequence (flip angle = 70°, repetition time = 2,000 ms, echo time = 30 ms, field of view = 210 mm, 64 × 64 matrix). Thirty-six descending axial slices (thickness = 3.0 mm, interslice gap = 0.3 mm) were acquired. Additionally, a high-resolution (1- × 1- × 1.2-mm) structural scan was acquired using a T1-weighted magnetization-prepared rapid-acquisition gradient-echo sequence. Participants 1 to 16 were scanned with an Achieva 3 Tesla scanner (Philips Medical Systems, Best, The Netherlands) using an eight-channel head coil. The remaining participants were scanned with a Magnetom Trio 3 Tesla scanner (Siemens, Erlangen, Germany) using a 12-channel head coil.

For preprocessing and statistical analysis, we used Statistical Parametric Mapping software (SPM8; Wellcome Trust Centre for Neuroimaging, London, United Kingdom; www.fil.ion.ucl.ac.uk/spm/) running in a MATLAB environment (Version 7.6; The MathWorks, Natick, MA). Preprocessing steps for the functional images included realigning and unwarping of the images to correct for head motion during the scan and slice-time correction. Images were normalized into a common space with the help of the high-resolution structural image. Using the VBM8 toolbox (http://dbm.neuro.uni-jena.de/vbm8), we (a) segmented the structural image into gray matter, white matter, and cerebrospinal fluid; (b) denoised the image; and (c) warped the image into the Montreal Neurological Institute (MNI) standard space using the high-dimensional DARTEL registration algorithm (Ashburner, 2007). Additionally, a skull-stripped version of the structural image was created in native space. The functional images were (a) coregistered to the skull-stripped structural image and (b) normalized to the MNI standard space using the parameters from the DARTEL registration of the structural image. Finally, the functional images were resampled to 2- × 2- × 2-mm voxels and smoothed with a 6-mm full-width half-maximum Gaussian kernel.

Statistical analysis of the fMRI data was performed within a two-stage mixed-effects model. In the first level (i.e., subject-specific level), we built a general linear model (Henson, 2004) including one regressor per item type (i.e., uppercase nouns, lowercase nouns, uppercase nonnouns, lowercase nonnouns, uppercase pseudowords, lowercase pseudowords). The regressors consisted of the trial onsets of the corresponding item type modeled by a stick function convolved with a synthetic hemodynamic response function. Additionally, six covariates corresponding to the movement parameters (rotations and translations) were included. The functional imaging data in these first-level models were high-pass filtered with a cutoff of 128 s and corrected for autocorrelation by an AR(1) model (Friston et al., 2002). For each participant, we computed contrast images reflecting signal change for each item type relative to fixation baseline (i.e., ISIs and null trials). These images were then used for the second-level (i.e., group-level) random-effects analysis. For statistical comparisons on the group level, we used a voxelwise threshold of *p* < .001 with an additional cluster extent threshold of *p* < .05, corrected for multiple comparisons using the family-wise error rate. To control for scanner-specific effects, we included the between-subjects factor scanner (Philips Achieva vs. Siemens Magnetom) in all group-level analyses reported in the Results. However, results for this factor are not reported because there were no significant interactions between scanner and any of the within-subjects factors of interest.

## Results

### Behavioral results

The present lexical decision task posed little difficulty for participants; there was an average of 95% correct responses across all trials. Participants were more accurate in correctly rejecting pseudowords (*M* = 98%) than correctly accepting words (*M* = 94%, pooled across nouns and nonnouns), *t*(25) = 4.99, *p* < .001, but were faster when responding to words relative to pseudowords, *t*(25) = 8.38, *p* < .001.

**Fig. 1.**
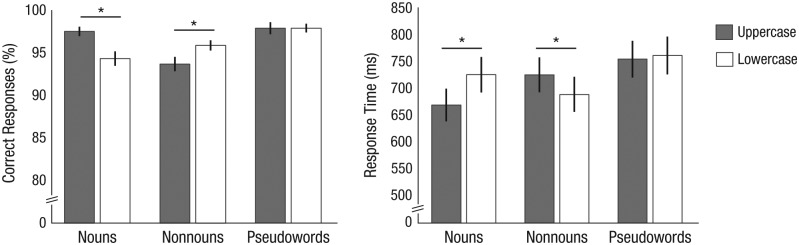
Behavioral results. Mean accuracy (left) and response time (right) in the lexical decision task are shown as a function of word type and case format. Error bars denote ±1 *SEM*. Asterisks indicate significant differences between case formats (*p* < .05).

Repeated measures 2 (word type: nouns vs. nonnouns) × 2 (case format: uppercase vs. lowercase) analyses of variance (ANOVAs) for the word items showed an interaction between the two factors for both accuracy, *F*(1, 25) = 7.28, *p* = .012, and response times (RTs), *F*(1, 25) = 85.67, *p* < .001. For nouns, higher accuracies, *t*(25) = 2.40, *p* = .024, and shorter RTs, *t*(25) = 9.57, *p* < .001, were found for the familiar uppercase format relative to the unfamiliar lowercase format. For nonnouns, higher accuracies, *t*(25) = 2.13, *p* = .043, and shorter RTs, *t*(25) = 4.25, *p* < .001, were found for the familiar lowercase format relative to the unfamiliar uppercase format. Paired-samples *t* tests for the pseudoword items showed no significant effect of the case format of the initial letter on accuracy, *t*(25) < 1, *p* > .250, or RTs, *t*(25) = 1.33, *p* = .195. Figure 1 presents mean accuracy and RT for all conditions of the study. RT analyses were based on trials with correct responses only.

### fMRI results

**Fig. 2.**
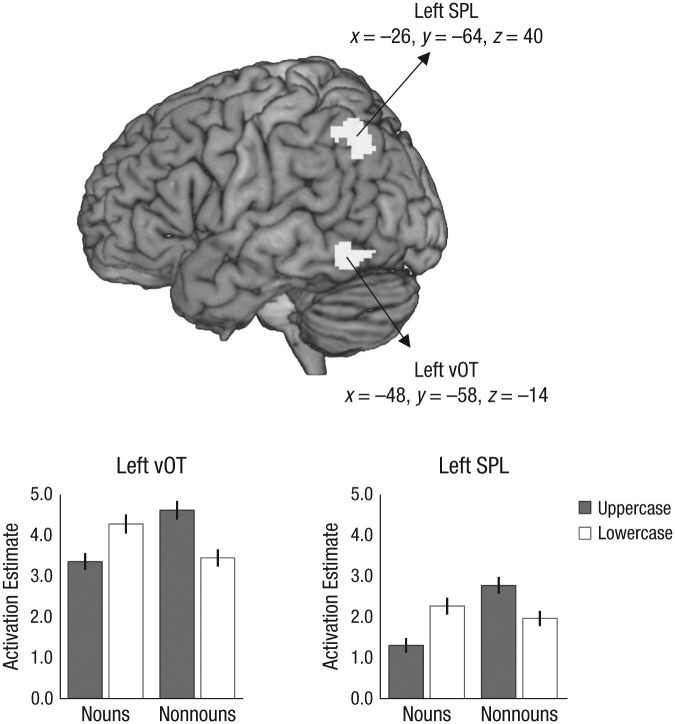
Functional MRI results. The brain map shows activation clusters identified by the Word Type (nouns vs. nonnouns) × Case Format (uppercase vs. lowercase) interaction for word items. The activation clusters, in left ventral occipitotemporal cortex (vOT) and left superior parietal lobule (SPL), are superimposed on a lateral view of the left hemisphere. The graphs show mean brain-activation estimates (in arbitrary units) as a function of word type and case format, separately for each cluster (peaks are given in Montreal Neurological Institute coordinates). Estimates were obtained relative to fixation baseline. Error bars denote ±1 *SEM*.

Of main interest for our hypothesis was the identification of brain regions with a differing response to the case format of the initial letter between nouns and nonnouns. To this end, we performed a 2 (word type) × 2 (case format) ANOVA on brain activation for the word items. To avoid differences arising from deactivations, we masked the analysis with a words > fixation baseline contrast (*p* < .001). Significant Word Type × Case Format interaction effects on brain activation were identified in two regions: the left vOT and the left superior parietal lobule (SPL; see Fig. 2 and Table 2). As can be seen from the plots in Figure 2, the activation patterns were similar in both clusters. For nouns, higher activation was found for items with initial letters in lowercase relative to items with initial letters in uppercase. The opposite pattern was found for nonnouns (i.e., uppercase > lowercase). Follow-up *t* tests confirmed that significant case-format differences (at least *p* < .001) were present for both word types in both clusters (see Table 2). The Word Type × Case Format interaction effect corresponds to a main effect of case-format familiarity (unfamiliar > familiar) independent of physical case format.

**Table 2.** Functional MRI Results: Brain Regions Showing a Word Type (Nouns vs. Nonnouns) × Case Format (Uppercase vs. Lowercase) Interaction Effect

		Peak MNI coordinates			Word Type × Case Format interaction: *F*(1, 75)	Uppercase > lowercase: *t*(25)		
Region	*k*	*x*	*y*	*z*		Nouns	Nonnouns
Left ventral occipitotemporal cortex	126	−48	−58	−14	30.8	−3.5	4.5
Left superior parietal lobule	177	−26	−64	40	21.7	−3.6	3.4

Note: The two rightmost columns show peak *t* values for post hoc comparisons between case formats. *k* = number of significant voxels in the cluster; MNI = Montreal Neurological Institute.

Additional ANOVA findings were that nonnouns elicited higher activation than nouns in a cluster located in the right angular gyrus (peak: *x* = 38, *y* = −56, *z* = 44; peak *F*(1, 75) = 20.94; cluster extent = 205 voxels) and that words with the initial letter in uppercase elicited higher activation than words with the initial letter in lowercase in a cluster in the right lingual gyrus (peak: *x* = 12, *y* = −84, *z* = −10; peak *F*(1, 75) = 18.99; cluster extent = 37 voxels). The latter finding was, however, significant only at *p* < .001 (uncorrected).

In a separate analysis, we searched for brain regions exhibiting a case-format effect for the pseudoword items. As in the analysis of the word items, we identified higher activation for uppercase compared with lowercase pseudowords in the right lingual gyrus (peak: *x* = 14, *y* = −84, *z* = −10; peak *F*(1, 25) = 20.24; cluster extent = 127 voxels).

Finally, we compared activation between pseudowords and words (pooled across nouns and nonnouns). As Table 3 shows, higher activation for pseudowords relative to words was found in the left precentral gyrus and in the supplementary motor area. Higher activation for words compared with pseudowords was found in the left angular gyrus and the right supramarginal gyrus. No significant differences between activations for words and pseudowords were found in left vOT or left SPL.

**Table 3.** Functional MRI Results: Brain Regions Identified by the Main Effect of Item Type (Words vs. Pseudowords)

		Peak MNI coordinates			
Contrast and region	*k*	*x*	*y*	*z*	*F*(1, 25)
Pseudowords > words					
Left precentral gyrus	205	−54	−4	44	5.83
Left precentral gyrus	409	−34	−22	56	5.14
Left supplementary motor area	170	−2	4	56	5.08
Words > pseudowords					
Left angular gyrus	268	−32	−62	40	5.34
Right supramarginal gyrus	259	50	−40	42	6.62

Note: *k* = number of significant voxels in the cluster; MNI = Montreal Neurological Institute.

## Discussion

In the present study, we investigated the predominant assumption in neurocognitive research that visual word recognition rests on abstract neural representations for written letters and words in the VWFA in the left vOT (Dehaene & Cohen, 2011; Dehaene et al., 2005). Several previous findings had raised doubts about the abstractness of orthographic representations and suggested that they might still contain information about the visual format in which words are most often seen (e.g., Jacobs et al., 2008). The present study showed that a minor violation of the typical visual format of German words (i.e., presenting the initial letter in an unfamiliar case format) increased brain activation in a left vOT region corresponding to the classic localization of the VWFA (Cohen et al., 2000; Cohen et al., 2002). This finding stands in contrast to the view that activation in this region is invariant to the specific visual appearance of words (Dehaene & Cohen, 2011).

By manipulating the case format of the initial letter of both German nouns (always seen capitalized) and nonnouns (mostly seen in lowercase), we were able to investigate the effect of case-format familiarity on VWFA activation independent of visual factors (i.e., physical case format). This overcame the drawbacks of previous neuroimaging studies (Kronbichler et al., 2009; Xu et al., 2001) that found increased left vOT activation for unfamiliar mixed-case formats, which are also known to result in low-level visual difficulties (Mayall et al., 1997). In contrast to these findings, the present case-familiarity effect was restricted to the left vOT region corresponding to the VWFA and was not seen in more posterior regions. Therefore, the present effect in the VWFA cannot be interpreted as a downstream effect of high activation in occipital regions. We did, however, identify a right occipital region that exhibited higher activation for uppercase relative to lowercase letters. This finding is in line with previous research showing that physical characteristics of visual words, such as number of letters, affect activation in early visual regions (Mechelli, Humphreys, Mayall, Olson, & Price, 2000; Schurz et al., 2010).

Proponents of abstract representations in the VWFA have argued that increased activation in the region might be the result of top-down processes rather than a reflection of the nature of local representations (Dehaene & Cohen, 2011). For example, it could be argued that the unfamiliarity of the case format might be detected only after the instantiation of abstract representations in the VWFA at the level of grammatical processing (i.e., on the basis of capitalization rules such as “if noun, then uppercase”), which leads to a top-down reactivation of the VWFA. However, if this were the case, it should have also resulted in increased activation in brain regions associated with higher language processes. The finding that no increased activation to the unfamiliar case formats was observed in any temporal or frontal brain regions associated with language processing (Price, 2012) speaks against the concern that the observed increased activation in the VWFA was driven by higher language processes.

Another possible concern with the present findings is that because fMRI integrates the brain signal over a long period of time, increased VWFA activation could also reflect greater processing time (Dehaene & Cohen, 2011). Critically, unfamiliar case formats of words resulted not only in increased VWFA activation but also in longer RTs relative to familiar formats. However, the RT difference between unfamiliar and familiar case formats for words (*M* = 48.7 ms) was similar to the RT difference between pseudowords and words (pooled across nouns and nonnouns; *M* = 49.4 ms). If activation in the VWFA can be generally explained by processing time, one could have also expected increased activation for pseudowords relative to words. This was not the case. Even with a very lenient statistical threshold (*p* < .01), we did not observe higher VWFA activation for pseudowords relative to words. The observed RT difference between unfamiliar and familiar case formats should therefore be viewed as a behavioral index of the cognitive mechanism that also underlies the increased brain activation: the mismatch between stimulus (i.e., unfamiliar case formats) and stored word representation (for a general discussion of the relation between RT effects and brain activation, see Taylor, Rastle, & Davis, 2014).

In addition to the VWFA, a left SPL cluster (*x* = −26, *y* = −64, *z* = 40) also exhibited higher activation for unfamiliar than for familiar case formats. This region has generally been associated with (visual) attentional demands (e.g., Corbetta & Shulman, 2002; Corbetta, Shulman, Miezin, & Petersen, 1995). With respect to visual word processing, Cohen, Dehaene, Vinckier, Jobert, and Montavont (2008) found increased SPL activation when words were spatially distorted by abnormal letter spacing or by nonhorizontal displays and required serial movement of attention. Furthermore, Vinckier et al. (2006) described a patient with a left parietal lesion who exhibited severe reading impairment when words were presented in the attentionally demanding displays of Cohen et al. (2008) but no impairment for words presented in the familiar format. On the basis of these findings, we suggest that the increased left SPL activation we found to the unfamiliar case formats of words might reflect an attentional response when a mismatch between stimulus (e.g., *ball*) and stored word representation (e.g., *Ball*) is registered in the VWFA.

Because the case format of the initial letter is a characteristic of whole words, the present findings support the view that the VWFA hosts representations for whole words (Glezer, Jiang, & Riesenhuber, 2009; Kronbichler et al., 2004; Ludersdorfer, Schurz, Richlan, Kronbichler, & Wimmer, 2013) and thus might serve as an orthographic lexicon, as posited by dual-route models of reading (e.g., Coltheart, Rastle, Perry, Langdon, & Ziegler, 2001). Support for this view also comes from previous studies that found increased VWFA activation for unfamiliar compared with familiar spellings of the same phonological words (e.g., *brane* vs. *brain*; Kronbichler et al., 2007; Kronbichler et al., 2009).

In conclusion, the findings of the present study suggest that neural representations of written words in the VWFA contain information about the visual format in which words are most frequently perceived. Such a grounding of memory representations in visual perception is denied by current neuroscientific models of visual word recognition (Dehaene et al., 2005), which assume that these representations are abstract and thus invariant to visual characteristics, such as font or case. However, the fact that visual word recognition is robust enough to deal with even very unfamiliar formats (e.g., *fbi* for *FBI*) does not necessarily speak against representations preserving the most frequently encountered appearance.

## References

[bibr1-0956797616657319] Ashburner J. (2007). A fast diffeomorphic image registration algorithm. NeuroImage, 38, 95–113. 17761438 10.1016/j.neuroimage.2007.07.007

[bibr2-0956797616657319] Baayen R. H. Piepenbrock R. van Rijn H. R. (1993). The CELEX lexical database [CD-ROM]. Philadelphia, PA: Linguistic Data Consortium.

[bibr3-0956797616657319] Burgund E. D. Guo Y. Aurbach E. L. (2009). Priming for letters and pseudoletters in mid-fusiform cortex: Examining letter selectivity and case invariance. Experimental Brain Research, 193, 591–601. 19039579 10.1007/s00221-008-1661-9

[bibr4-0956797616657319] Carreiras M. Perea M. Gil-López C. Mallouh R. A. Salillas E. (2013). Neural correlates of visual versus abstract letter processing in Roman and Arabic scripts. Journal of Cognitive Neuroscience, 25, 1975–1985. 23806176 10.1162/jocn_a_00438 PMC3837287

[bibr5-0956797616657319] Cohen L. Dehaene S. Naccache L. Lehéricy S. Dehaene-Lambertz G. Hénaff M. A. Michel F. (2000). The visual word form area: Spatial and temporal characterization of an initial stage of reading in normal subjects and posterior split-brain patients. Brain, 123, 291–307. 10648437 10.1093/brain/123.2.291

[bibr6-0956797616657319] Cohen L. Dehaene S. Vinckier F. Jobert A. Montavont A. (2008). Reading normal and degraded words: Contribution of the dorsal and ventral visual pathways. NeuroImage, 40, 353–366. 18182174 10.1016/j.neuroimage.2007.11.036

[bibr7-0956797616657319] Cohen L. Lehéricy S. Chochon F. Lemer C. Rivaud S. Dehaene S. (2002). Language-specific tuning of visual cortex? Functional properties of the visual word form area. Brain, 125, 1054–1069. 11960895 10.1093/brain/awf094

[bibr8-0956797616657319] Coltheart M. Rastle K. Perry C. Langdon R. Ziegler J. (2001). DRC: a dual route cascaded model of visual word recognition and reading aloud. Psychological Review, 108, 204–256. 11212628 10.1037/0033-295x.108.1.204

[bibr9-0956797616657319] Corbetta M. Shulman G. L. (2002). Control of goal-directed and stimulus-driven attention in the brain. Nature Reviews Neuroscience, 3, 201–215. 11994752 10.1038/nrn755

[bibr10-0956797616657319] Corbetta M. Shulman G. L. Miezin F. M. Petersen S. E. (1995). Superior parietal cortex activation during spatial attention shifts and visual feature conjunction. Science, 270, 802–805. 7481770 10.1126/science.270.5237.802

[bibr11-0956797616657319] Dehaene S. Cohen L. (2011). The unique role of the visual word form area in reading. Trends in Cognitive Sciences, 15, 254–262. 21592844 10.1016/j.tics.2011.04.003

[bibr12-0956797616657319] Dehaene S. Cohen L. Sigman M. Vinckier F. (2005). The neural code for written words: A proposal. Trends in Cognitive Sciences, 9, 335–341. 15951224 10.1016/j.tics.2005.05.004

[bibr13-0956797616657319] Dehaene S. Jobert A. Naccache L. Ciuciu P. Poline J. B. Le Bihan D. Cohen L. (2004). Letter binding and invariant recognition of masked words: Behavioral and neuroimaging evidence. Psychological Science, 15, 307–313. 15102139 10.1111/j.0956-7976.2004.00674.x

[bibr14-0956797616657319] Dehaene S. Naccache L. Cohen L. Le Bihan D. Mangin J. F. Poline J. B. Rivière D. (2001). Cerebral mechanisms of word masking and unconscious repetition priming. Nature Neuroscience, 4, 752–758. 11426233 10.1038/89551

[bibr15-0956797616657319] Devlin J. T. Jamison H. L. Gonnerman L. M. Matthews P. M. (2006). The role of the posterior fusiform gyrus in reading. Journal of Cognitive Neuroscience, 18, 911–922. 16839299 10.1162/jocn.2006.18.6.911 PMC1524880

[bibr16-0956797616657319] Friston K. J. Glaser D. E. Henson R. N. Kiebel S. Phillips C. Ashburner J. (2002). Classical and Bayesian inference in neuroimaging: Applications. NeuroImage, 16, 484–512. 12030833 10.1006/nimg.2002.1091

[bibr17-0956797616657319] Gaillard R. Naccache L. Pinel P. Clémenceau S. Volle E. Hasboun D.. . . Cohen L. (2006). Direct intracranial, fMRI, and lesion evidence for the causal role of left inferotemporal cortex in reading. Neuron, 50, 191–204. 16630832 10.1016/j.neuron.2006.03.031

[bibr18-0956797616657319] Gauthier I. Tarr M. J. Moylan J. Skudlarski P. Gore J. C. Anderson A. W. (2000). The fusiform “face area” is part of a network that processes faces at the individual level. Journal of Cognitive Neuroscience, 12, 495–504. 10931774 10.1162/089892900562165

[bibr19-0956797616657319] Glezer L. S. Jiang X. Riesenhuber M. (2009). Evidence for highly selective neuronal tuning to whole words in the “visual word form area.” Neuron, 62, 199–204. 19409265 10.1016/j.neuron.2009.03.017 PMC2706007

[bibr20-0956797616657319] Henson R. N. A. (2004). Analysis of fMRI time series: Linear time-invariant models, event-related fMRI and optimal experimental design. In Frackowiak R. S. J. Friston K. J. Frith C. Dolan R. Price C. J. Zeki S. Ashburner J. T. Penny W. D. (Eds.), Human brain function (2nd ed., pp. 793–822). London, England: Academic Press.

[bibr21-0956797616657319] Jacobs A. M. Nuerk H. C. Graf R. Braun M. Nazir T. A. (2008). The initial capitalization superiority effect in German: Evidence for a perceptual frequency variant of the orthographic cue hypothesis of visual word recognition. Psychological Research, 72, 657–665. 18841389 10.1007/s00426-008-0168-0

[bibr22-0956797616657319] Kronbichler M. Bergmann J. Hutzler F. Staffen W. Mair A. Ladurner G. Wimmer H. (2007). Taxi vs. taksi: On orthographic word recognition in the left ventral occipitotemporal cortex. Journal of Cognitive Neuroscience, 19, 1584–1594. 17933023 10.1162/jocn.2007.19.10.1584 PMC2989180

[bibr23-0956797616657319] Kronbichler M. Hutzler F. Wimmer H. Mair A. Staffen W. Ladurner G. (2004). The visual word form area and the frequency with which words are encountered: Evidence from a parametric fMRI study. NeuroImage, 21, 946–953. 15006661 10.1016/j.neuroimage.2003.10.021

[bibr24-0956797616657319] Kronbichler M. Klackl J. Richlan F. Schurz M. Staffen W. Ladurner G. Wimmer H. (2009). On the functional neuroanatomy of visual word processing: Effects of case and letter deviance. Journal of Cognitive Neuroscience, 21, 222–229. 18476755 10.1162/jocn.2009.21002 PMC2976854

[bibr25-0956797616657319] Leff A. P. Crewes H. Plant G. T. Scott S. K. Kennard C. Wise R. J. S. (2001). The functional anatomy of single-word reading in patients with hemianopic and pure alexia. Brain, 124, 510–521. 11222451 10.1093/brain/124.3.510

[bibr26-0956797616657319] Ludersdorfer P. Schurz M. Richlan F. Kronbichler M. Wimmer H. (2013). Opposite effects of visual and auditory word-likeness on activity in the visual word form area. Frontiers in Human Neuroscience, 7, Article 491. doi: 10.3389/fnhum.2013.00491 PMC3756304 24009569

[bibr27-0956797616657319] Mayall K. Humphreys G. W. Olson A. (1997). Disruption to word or letter processing? The origins of case-mixing effects. Journal of Experimental Psychology: Learning, Memory, and Cognition, 23, 1275–1286. 10.1037//0278-7393.23.5.1275 9293635

[bibr28-0956797616657319] Mechelli A. Humphreys G. W. Mayall K. Olson A. Price C. J. (2000). Differential effects of word length and visual contrast in the fusiform and lingual gyri during reading. Proceedings of the Royal Society B: Biological Sciences, 267, 1909–1913. 10.1098/rspb.2000.1229 PMC1690747 11052544

[bibr29-0956797616657319] Peressotti F. Cubelli R. Job R. (2003). On recognizing proper names: The orthographic cue hypothesis. Cognitive Psychology, 47, 87–116. 12852936 10.1016/s0010-0285(03)00004-5

[bibr30-0956797616657319] Price C. J. (2012). A review and synthesis of the first 20 years of PET and fMRI studies of heard speech, spoken language and reading. NeuroImage, 62, 816–847. 22584224 10.1016/j.neuroimage.2012.04.062 PMC3398395

[bibr31-0956797616657319] Price C. J. Devlin J. T. (2003). The myth of the visual word form area. NeuroImage, 19, 473–481. 12880781 10.1016/s1053-8119(03)00084-3

[bibr32-0956797616657319] Price C. J. Devlin J. T. (2011). The interactive account of ventral occipitotemporal contributions to reading. Trends in Cognitive Sciences, 15, 246–253. 21549634 10.1016/j.tics.2011.04.001 PMC3223525

[bibr33-0956797616657319] Rayner K. Pollatsek A. Ashby J. Clifton C. Jr. (2012). Psychology of reading (2nd ed.). New York, NY: Psychology Press.

[bibr34-0956797616657319] Schurz M. Sturm D. Richlan F. Kronbichler M. Ladurner G. Wimmer H. (2010). A dual-route perspective on brain activation in response to visual words: Evidence for a length by lexicality interaction in the visual word form area (VWFA). NeuroImage, 49, 2649–2661. 19896538 10.1016/j.neuroimage.2009.10.082 PMC2989181

[bibr35-0956797616657319] Taylor J. S. H. Rastle K. Davis M. H. (2014). Interpreting response time effects in functional imaging studies. NeuroImage, 99, 419–433. 24904992 10.1016/j.neuroimage.2014.05.073 PMC4121088

[bibr36-0956797616657319] Vinckier F. Dehaene S. Jobert A. Dubus J. P. Sigman M. Cohen L. (2007). Hierarchical coding of letter strings in the ventral stream: dissecting the inner organization of the visual word-form system. Neuron, 55, 143–156. 17610823 10.1016/j.neuron.2007.05.031

[bibr37-0956797616657319] Vinckier F. Naccache L. Papeix C. Forget J. Hahn-Barma V. Dehaene S. Cohen L. (2006). “What” and “where” in word reading: Ventral coding of written words revealed by parietal atrophy. Journal of Cognitive Neuroscience, 18, 1998–2012. 17129187 10.1162/jocn.2006.18.12.1998

[bibr38-0956797616657319] Xu B. Grafman J. Gaillard W. D. Ishii K. Vega-Bermudez F. Pietrini P.. . . Theodore W. (2001). Conjoint and extended neural networks for the computation of speech codes: The neural basis of selective impairment in reading words and pseudowords. Cerebral Cortex, 11, 267–277. 11230098 10.1093/cercor/11.3.267

